# Organization of Neural Systems for Aversive Information Processing: Pain, Error, and Punishment

**DOI:** 10.3389/fnins.2012.00136

**Published:** 2012-09-21

**Authors:** Shunsuke Kobayashi

**Affiliations:** ^1^Department of Neurology, Fukushima Medical UniversityFukushima, Japan

**Keywords:** amygdala, periaqueductal gray, orbitofrontal cortex, anterior cingulate cortex, prefrontal cortex, error-related negativity, pain, reward

## Abstract

The avoidance of aversive events is critically important for the survival of organisms. It has been proposed that the medial pain system, including the amygdala, periaqueductal gray (PAG), and anterior cingulate cortex (ACC), contains the neural circuitry that signals pain affect and negative value. This system appears to have multiple defense mechanisms, such as rapid stereotyped escape, aversive association learning, and cognitive adaptation. These defense mechanisms vary in speed and flexibility, reflecting different strategies of self-protection. Over the course of evolution, the medial pain system appears to have developed primitive, associative, and cognitive solutions for aversive avoidance. There may be a functional grading along the caudal-rostral axis, such that the amygdala-PAG system underlies automatic and autonomic responses, the amygdala-orbitofrontal system contributes to associative learning, and the ACC controls cognitive processes in cooperation with the lateral prefrontal cortex. A review of behavioral and physiological studies on the aversive system is presented, and a conceptual framework for understanding the neural organization of the aversive avoidance system is proposed.

## Introduction

The nervous system has multiple mechanisms for protecting organisms against harmful events. Reflexes in the spinal cord and brainstem provide the most primitive form of defense, such as withdrawing a hand upon touching a hot object. Association learning is a higher mechanism that allows organisms to anticipate harmful events. Since the aversive consequences may be damaging or even fatal, organisms cannot afford many exposures thereto, and aversive learning must be sufficiently fast. Harmful events may be avoided by cognitive functions such as performance monitoring, error detection, and top-down attention control.

**Figure 1:**
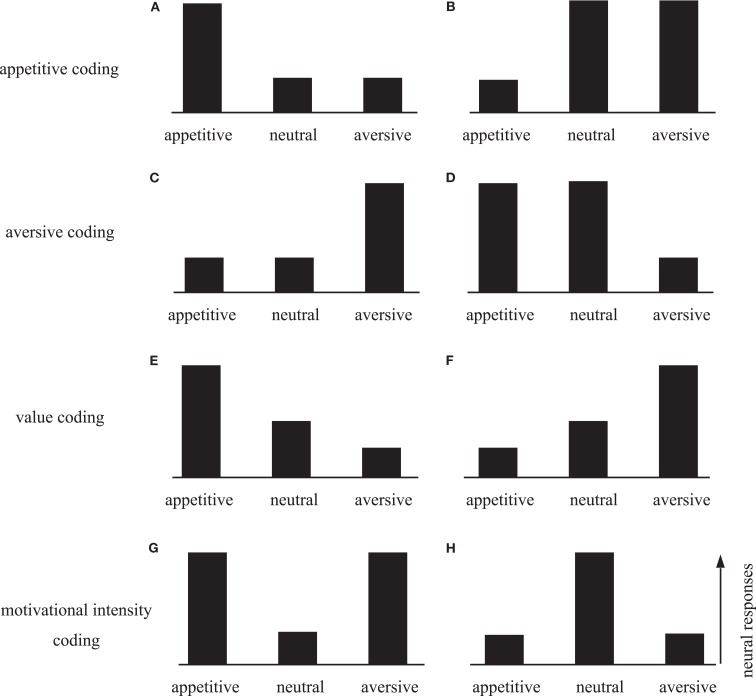
**Schematic diagram of the neural response patterns to appetitive, neutral, and aversive stimuli**. Neurons that selectively process appetitive information would be selectively activated **(A)** or suppressed **(B)** by appetitive stimuli. In this case, the responses to aversive and neutral stimuli would not differ. Similarly, neurons in the aversive system would exhibit selective activation **(C)** or suppression **(D)** to aversive stimuli. Single neurons may encode both appetitive and aversive information on the value scale, such that high activity reflects a positive (appetitive) value and low activity reflects a negative (aversive) value **(E)**, or vice versa **(F)**. Neurons may encode motivational intensity independent of valence. Neural responses may be enhanced **(G)** or suppressed **(H)** by motivationally significant stimuli regardless of whether they are appetitive or aversive. These types of responses may be related to attention or arousal level.

This paper discusses the neural mechanisms underlying aversive avoidance, focusing on two aspects. First, it may be important to understand how the neural system implements aversive avoidance. Because it is so critical for survival, the avoidance system must have developed under great evolutionary pressure. There seem to be multiple avoidance mechanisms reflecting different evolutionary stages. Thus, understanding the neural organization of the aversive system may provide insight into its evolution and development. Second, the aversive system is an essential counterpart of the reward system. An important issue is thus how the brain processes the information of opposing valences. In theory, rewarding and aversive events can be encoded on one scale in the positive and negative ranges, respectively. Alternatively, events of the opposite valences may be processed by distinct neural networks. Figure 1 illustrates the possible encoding of rewarding and aversive events, where the bars indicate hypothetical neural activities in response to appetitive, motivationally neutral, and aversive events. Preferential excitation or suppression of appetitive events with reference to neutral events indicates that neurons are sensitive to positive value (Figures 1A,B). Conversely, preferential excitation or suppression to aversive events indicates a negative value coding (Figures 1C,D). Single neurons may encode both positive and negative ranges on the value scale, such that high activity reflects positive (appetitive) value and low activity reflects negative (aversive) value (Figure 1E), or vice versa (Figure 1F). Another possibility is that neurons respond to both appetitive and aversive events in the same direction, but not to neutral events (Figures 1G,H). This type of response encodes motivational intensity, possibly reflecting the level of attention or arousal.

## Perception of Aversive Stimuli

**Figure 2:**
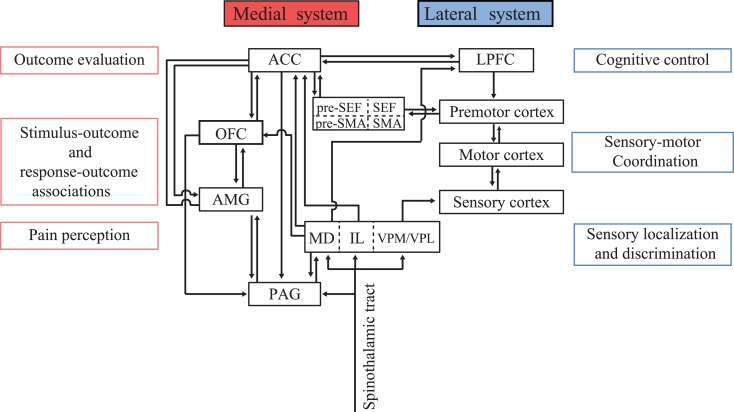
**Conceptual schema illustrating the functional dichotomy between the medial and lateral systems**. The medial system includes the amygdala (AMG), periaqueductal gray (PAG), orbitofrontal cortex (OFC), and anterior cingulate cortex (ACC), and serves as a predictor and evaluator of behavioral outcomes. The lateral system receives multimodal sensory inputs and processes the signals to obtain physical information regarding the environment. Sensory information is transferred to the LPFC for cognitive decision-making and action planning. The ACC provides feedback information to the LPFC to implement behavioral adaptation based on outcome values. MD, mediodorsal nucleus of the thalamus; IL, intralaminar nucleus of the thalamus; VPM/VPL, ventral posteromedial nucleus/ventral posterolateral nucleus of the thalamus; SMA, supplementary motor area; pre-SMA, presupplementary motor area; LPFC, lateral prefrontal cortex.

The perception of aversive stimuli is crucial for the survival of organisms. Noxious stimuli applied to the skin activate various brain areas including the thalamus, primary somatosensory cortex (S1), anterior insular cortex, periaqueductal gray (PAG), amygdala, and anterior cingulate cortex (ACC; Jones et al., 1991; Talbot et al., 1991; Coghill et al., 1994; Hutchison et al., 1999; Koyama et al., 2001; Iwata et al., 2005). Thus, nociceptive input is processed in distributed sensory networks. Previous studies have suggested the presence of a crude dichotomy of sensory processing, namely into the lateral cortical pathway for sensory localization and discrimination, and the medial subcortical-limbic pathway (or medial pain system) for processing affective and motivational significance based on sensory information (Figure 2; Vogt et al., 1993a; Schnitzler and Ploner, 2000; Vogt and Sikes, 2000; Zhang et al., 2011).

The medial pain system includes the medial and intralaminar thalamic nuclei, ACC, and projections from these areas to nociception-regulating centers such as the PAG (Vogt et al., 1993b). The PAG receives inputs from the axon collaterals of spinothalamic projections and is connected reciprocally with the medial thalamic nuclei and the central nucleus of the amygdala. The PAG has been implicated as a key player in the descending noxious inhibitory system (Le Bars et al., 1979). The involvement of the PAG in pain control has been demonstrated by analgesic effects caused by opiate injection and electrical stimulation in the PAG (Mayer and Liebeskind, 1974; Bennett and Mayer, 1979; Yaksh et al., 1988).

The ACC is linked to the medial pain system via its medial thalamic afferents and projections to the PAG. The ACC is thought to receive nociceptive inputs from the medial and intralaminar thalamic nuclei (Hsu and Shyu, 1997). Nociceptive neurons in the ACC have characteristically extensive dendritic arbors in layer IIIc, where the thalamic projection terminates (Vogt et al., 1981). Complete disconnection of ACC from the S1 does not abolish nociceptive responses in the ACC, which indicates that nociceptive responses in the ACC are independent of those in the S1 (Sikes and Vogt, 1992). High-density opiate receptors in the ACC also support its role in pain perception. Neural populations in the ACC responds to dermal stimulation by noxious CO_2_ laser with short- and long-latency components of possibly A-δ and C-fiber origins, respectively. Intraperitoneal administration of morphine significantly attenuates both of these components in the ACC (Kuo and Yen, 2005). It has been proposed that the phasic nociceptive responses in the ACC are mediated by the thalamus, and the long-duration responses may underlie integrative processes following the primary thalamic-mediated nociceptive responses (Shyu et al., 2010). It is also known that cingulate lesions reduce affective responses to noxious stimuli without disrupting sensory localization (Foltz and White, 1962; Ballantine et al., 1967).

A study on rabbits examined the neuronal responses to visceral pain caused by balloon distension applied to the colon and cutaneous pain caused by thermal and electrical stimuli applied to the skin (Sikes et al., 2008). A group of ACC neurons exhibited a viscerocutaneous response (39.1%), while others were exclusively visceral (37.3%) or exclusively cutaneous (22.6%). That study also found that the nociceptive response was not strictly limited to the ACC, with pain being more extensively represented in the medial frontal area including midcingulate and retrosplenial cortices.

The medial pain system may also be involved in the motor and autonomic responses induced by aversive stimuli. For example, the freezing response to electric shock is thought to be elicited via the amygdala-PAG pathway in rats (Ledoux et al., 1988; Amorapanth et al., 2000) and cats (Hopkins and Holstege, 1978; Amorapanth et al., 2000), although its course downstream from the PAG remains unclear. The medial raphe nucleus is also involved in freezing and other anxiety-related responses, such as increased micturition, defecation, crouching, and piloerection (Graeff and Silveira Filho, 1978). Lesioning the medial raphe nucleus suppressed fear-induced behaviors but relatively preserved simple appetitive behaviors.

It is debatable whether the medial pain system responds preferentially to aversive stimuli or commonly to both rewarding and aversive stimuli. Few studies have examined the ACC responses to both appetitive and aversive stimuli. A primate single-unit study of the amygdala demonstrated the coexistence of both valence-sensitive and valence-insensitive neurons; some amygdala neurons exhibited differential responses to rewards only, others to punishments only, and some neurons to both rewards and punishments (Belova et al., 2007). Responses to appetitive and aversive stimuli appear to change according to context in both the amygdala and ACC. For example, the response to juice appears to differ markedly according to whether juice delivery is predicted (Koyama et al., 2001; Belova et al., 2007). Therefore, the sensory response of the ACC may not simply reflect sensory input *per se*, instead also being influenced by top-down modulation.

Harmful events may be perceived not only through somatosensory inputs but also through other sensory modalities, including odor and gustatory sensations. A question arises as to whether the neural system generates a generic aversive signal that does not depend upon a specific input modality. Neuroimaging studies have investigated the brain structures that are commonly activated by different modalities of aversive stimuli (e.g., aversive pictures and uncomfortable temperatures). Common aversive responses were found in the amygdala, anterior insular cortex, orbitofrontal cortex (OFC), and ACC (Hayes and Northoff, 2011). Together, these findings suggest that the medial pain system signals the generic negative affects induced by multiple sensory modalities.

## Neural Correlates of Aversive Association Learning

For wild animals, the presence of a predator’s odor or footprints indicates impending danger and learning aversive associations is critically important for their survival. In the laboratory, rodents exhibit excitatory or inhibitory responses (i.e., startle, escape, and freezing) to an innocuous stimulus (i.e., a tone) that predicts a noxious stimulus (i.e., electric shock), and amygdala lesions cause behavioral impairments in these aversive conditioning (Pellegrino, 1968; Slotnick, 1973; Wilensky et al., 1999; Davis et al., 2003; Blair et al., 2005). Primate studies have also demonstrated behavioral impairments after amygdala lesions related to aversive avoidance, such as consuming unpleasant foods or the avoidance of predators or unfriendly conspecifics (Machado and Bachevalier, 2006; Machado et al., 2010).

Despite overwhelming evidence for the role of the amygdala in fear conditioning, neural signaling of aversive learning remains largely unclear. As for appetitive learning, reward prediction error theory has been proposed as a mechanism underlying the actions of the dopamine system, which enables associations between conditioned stimuli (CS) and appetitive unconditioned stimuli (US). According to that theory, behavioral adaptation is guided by a teaching signal that reflects the gap between predicted and actual reward outcomes. Most dopamine neurons reflect the reward prediction error; while animals learn the associations between CS (e.g., a picture) and US (e.g., juice), the initially present dopamine activations to appetitive US disappear and responses to CS emerge (Ljungberg et al., 1992; Schultz et al., 1997). Few studies have examined whether the prediction error theory applies to neural activities during aversive conditioning. Dopamine response during aversive learning may be a mirror image of that during reward learning, such that initial suppressions to aversive US disappear and suppressions to aversive CS emerge (Mirenowicz and Schultz, 1996; Matsumoto and Hikosaka, 2009; Cohen et al., 2012). Johansen et al. (2010) examined the influences of prediction on neural responses in the amygdala and PAG during fear conditioning. Unpredicted shock-evoked responses in both the amygdala and PAG, but these responses diminished when shock was predicted by CS. Furthermore, pharmacological inactivation of the PAG attenuated the shock-evoked responses in the amygdala and impaired acquisition of fear conditioning. Another study found that CS responses in amygdala neurons emerged during fear conditioning (Quirk et al., 1995). These results suggest that prediction error theory applies to the process of aversive learning in the dopamine system, amygdala, and PAG.

Another issue is whether the amygdala specializes in aversive conditioning in a valence-selective manner. Recent studies have suggested that the amygdala is involved in the behavioral responses to both appetitive and aversive reinforcements. Paton et al. (2006) recorded from amygdala neurons while abstract images acquired positive and negative values during conditioning with a liquid reward and air-puff, respectively. They found that distinct populations of amygdala neurons encode the positive and negative values of visual stimuli and that changes in neuronal activity correlated with the behavioral responses of anticipatory licking and eye blinking. It has also been found that some amygdala neurons are activated by both rewarding and aversive stimuli in the same direction, which may reflect the level of arousal or attention (Belova et al., 2007). Thus, although classically viewed as a center of fear, the amygdala seems to represent opponent motivational valences as well as motivational intensity.

It has also been suggested that the OFC is involved in association learning and value representation. Gottfried et al. (2002) used functional MRI (fMRI) in humans to study hemodynamic responses during odor-face conditioning, where initially neutral faces were repetitively paired with pleasant, neutral, or unpleasant odors. That study identified several key areas involved in olfactory associative learning, including the OFC, the nucleus accumbens, and the amygdala. Within the OFC, regions related to olfactory association learning were found rostral to the regions that show odor-evoked activity. Those authors demonstrated that olfactory input transforms from sensory to associative signals through caudal-to-rostral processing in the OFC. Other imaging studies have suggested the occurrence of compartmentalization of the opposing value signals within the OFC, with lateral activation in response to rewarding stimuli and medial activation in response to punishing stimuli (O’Doherty et al., 2001; Small et al., 2001).

Considering the extensive bidirectional anatomical connections, the amygdala and OFC are likely to have close functional interactions (Carmichael and Price, 1995; Morrison et al., 2011). With multimodal sensory afferents and the projections to the autonomic centers, the amygdala-OFC system is located strategically to underlie behavioral adaptation based on conditioning.

## Neural Basis of Error Detection and Behavioral Adaptation

Inappropriate behavior may lead to aversive outcomes. Such behavior may be suppressed through a process called operant conditioning, in which the associations between behavioral acts and their consequences are learned. Nevertheless, response errors may occur even after operant learning has progressed, such as in the presence of distraction, interference, or conflict. Empirical data show that subjects often recognize error commission and prepare for compensatory or defensive responses to upcoming aversive outcomes. One question is how error commission can be recognized before the associated negative outcomes are revealed.

Error-related negative (ERN) deflection of the EEG is probably the most replicated evidence for on-line neural processing of the occurrence of errors (Falkenstein et al., 1990). The ERNs have a symmetrical frontocentral distribution, and dipole modeling has consistently indicated that they originate from the medial frontal cortex, specifically in the ACC (Dehaene et al., 1994; Holroyd et al., 1998; Gehring et al., 2000). ERNs are elicited by incorrect responses in various tasks with different response modalities (e.g., hands, feet, and eyes; Holroyd et al., 1998; Nieuwenhuis et al., 2001). Miltner et al. (2003) required participants to press a button when they estimated that 1s had elapsed following presentation of a warning stimulus. At the end of the trial, a feedback stimulus indicated whether or not their estimate on that trial was within a criterion range. That study demonstrated that ERNs are elicited by error feedback, which was temporally dissociated with the occurrences of behavioral response. Other studies replicated this finding by presenting error feedback in the auditory, visual, and somatosensory modalities. Thus, the ERNs appear to reflect neural error processing that is flexible and generic in that it is triggered by either motor responses or error feedback and that it depends on the modality of neither the behavioral response nor the sensory feedback.

Experiencing error feedback is not uncommon in our daily lives (e.g., a cash dispenser giving a beep sound when invalid PIN is typed). Error feedback serves as a negative reinforcer because we adapt our behavior to avoid receiving such signals. Feedback signals that are contingent upon error responses are usually human inventions (e.g., beep sound, flashing LED). Thus, learning based on error feedback might be considered to be unique to humans. However, animals also show behavioral adaptation based on negative feedback during operant tasks in laboratories. In theory, the violation of a response-outcome contingency is associated with prediction error, which serves as a teaching signal to guide reinforcement learning. It has been suggested by some researchers that dopamine neurons are a potential origin of ERNs, because they are known to carry prediction error signals and project to the medial frontal cortex (Holroyd and Coles, 2002). Alternatively, the medial frontal cortex may supply prediction error signals via its connections to the midbrain dopamine area. The association with the dopamine system suggests that ERNs should be driven by unexpected positive (successes and rewards) and negative (errors and punishments) events in opposing directions (Figures 1E,F). This hypothesis is supported by empirical data, and in particular for negative prediction errors. Holroyd and Coles (2002) found that a larger ERN was elicited by unexpected unfavorable outcomes than by expected unfavorable outcomes, which indicates that ERNs are correlated more strongly with negative prediction errors than with the negative outcomes themselves. Other studies have suggested that event-related potentials of medial frontal origin respond particularly strongly to outcomes that are considered aversive or signaling reductions in reward (Bush et al., 2002; Holroyd and Coles, 2002; Nieuwenhuis et al., 2004).

Primate studies also support the idea that the medial frontal cortex is involved in error-related processing. In a series of studies (Schall et al., 2002) used a saccadic stop-signal task, in which saccades that were supposed to reach peripheral targets had to be canceled upon presentation of a stop-signal. Surface EEGs recorded in monkeys exhibited a greater negative deflection when saccades were not canceled on stop trials than when saccades were correctly executed on no-stop trials (Godlove et al., 2011). This monkey homolog of ERNs is distributed in medial frontal areas, similar to human ERNs. It has also been shown that local field potentials (LFPs) and single-unit activities in the monkey ACC and supplementary eye field (SEF) are modulated on error commission (Niki and Watanabe, 1979; Stuphorn et al., 2000; Ito et al., 2003; Emeric et al., 2008, 2010). ACC neurons that show post-error activations were also found to be active when the expected reward was omitted after correct behavior responses (Niki and Watanabe, 1979). Thus, ACC neurons may not be simply “error-related,” but may be reflecting negative reward prediction errors. On the other hand, LFPs in SEF were different from those in ACC in that they correlated with response conflict rather than reward prediction error (Emeric et al., 2008, 2010), suggesting that, unlike ACC, SEF is involved in sensory-motor processing.

In summary, there is growing evidence that ACC reflects prediction error in response-outcome contingencies. ERNs may be correlated more strongly with negative than with positive prediction errors (Chase et al., 2011), indicating predominantly aversive processing in the ACC (Figures 1C,D). Prediction error signals in the ACC may influence motor planning processes in the adjacent motor-related areas, including the SEF and supplementary motor area. Whether ERNs depend on dopamine input remains unclear; further investigation is needed in this field.

## Cognitive Control Theory and the ACC

While the prediction error hypothesis of ERNs implies value-based learning, value-independent theories have also been proposed as underlying mechanisms. Perhaps the most popular is the cognitive control theory, according to which ERNs reflect top-down attention control exerted with high cognitive demand, such as when there is an interfering stimulus to be ignored or a prepotent response to be inhibited. A typical situation is found in the Stroop task, in which subjects are required to name the print colors of color words (Stroop, 1935). When a word name and its print color are incongruent, the prepotent word-reading response must be inhibited and the print color has to be named. This conflict increases the rate of response errors and the reaction time. Human neuroimaging studies have consistently shown ACC activation on conflict trials during Stroop and other conflict tasks, including the Eriksen flanker (Gratton et al., 1992; Botvinick et al., 1999) and Simon tasks(Sturmer et al., 2002). However, primate neurophysiological studies have failed to find a conflict signal during tasks that should engender response conflict (Nakamura et al., 2005; Emeric et al., 2008). Future investigation should clarify whether the observed physiological differences in conflict paradigms are due to technical issues (e.g., differences in behavioral tasks and recording methods), or due to species heterogeneity of the ACC functions and cognitive flexibility (Cole et al., 2009). Different perspectives on the role of ACC (conflict theory versus outcome-based decision-making) might be reconciled by a modified theory of conflict monitoring (Botvinick, 2007).

It is known that prior context influences the size of the behavioral interference effects on subsequent trials during conflict tasks. An example is an increase in the behavioral reaction time following an error. Such post-error slowing indicates a reactive adjustment in cognitive control that shifts the speed-accuracy trade-off for more accurate responding (Rabbitt, 1966). Another type of sequential effects is a faster reaction time after conflict trials: the conflict effect decreases when the previous trial was incongruent compared to when the previous trial was congruent (Sturmer et al., 2002; Wuhr and Ansorge, 2005). This post-conflict behavioral adjustment is interpreted to be a result of top-down control recruited additionally by conflict on the previous trial. Consistent with this idea, fMRI studies have shown that conflict-related activity in the ACC is reduced after conflict trials (Botvinick et al., 1999; Kerns et al., 2004). Also, the post-conflict behavioral adjustment is attenuated in patients with medial frontal injuries (Di Pellegrino et al., 2007). Womelsdorf et al. (2010) found that LFPs recorded in the ACC while monkeys responded to a peripheral stimulus according to two stimulus-response (SR) mapping rules were selective for the SR mappings and stronger when behavioral adjustment was required following errors. These results suggest that the medial prefrontal cortex, and specifically the ACC, is involved in cognitive control based on conflict monitoring and error detection.

## Value-Based and Value-Independent Models for Behavioral Adjustment

Both reinforcement learning and cognitive control may guide decision-making and behavioral adaptation. However, they operate with different strategies and it has long been debated which strategy is implemented in the ACC and reflected in ERNs (Di Pellegrino et al., 2007). Reinforcement learning is a value-based algorithm, which would adjust behavioral output based on outcome evaluation. The dopamine and basal ganglia systems appear to operate under reinforcement learning algorithms. Thus, one possible hypothesis is that the medial pain system computes negative values based on reinforcement learning algorithms in parallel with reward computation in the dopamine and basal ganglia systems. In contrast, cognitive control theory is not directly concerned with outcome value. According to this theory, the ACC monitors cognitive demand and adjusts for allocation of cognitive resources. These two theories are not necessarily mutually exclusive: the mechanisms of reinforcement learning and cognitive control may coexist or cooperate in the ACC (Botvinick, 2007). Notably, the ACC is thought to be subdivided into areas of affects and cognition (Vogt, 1993; Devinsky et al., 1995). The affective division encompasses areas 25, 24, and 33, which have extensive connections with the amygdala and PAG. The cognitive division includes caudal areas 24′, 32′, and the cingulate motor areas, and the nociceptive cortex. Thus, heterogeneous functions may occur in different parts of the ACC.

## Influence of Outcome Value on Cognitive Processing

Environmental information is received as sensory input and its various physical features are processed in the cortical sensory areas. On the other hand, ventromedial brain structures, including the dopamine system, amygdala, OFC, and ACC, appear to play a key role in mapping sensory-motor information onto the scale of value (Figure 2). Given this functional dichotomy, how are decisions and action planning affected by the associated reward and punishment? There must be an interaction between the dorsolateral cognitive pathway and the ventromedial value pathway. Indeed, the influence of reward expectation on neuronal activities in various cortical areas has been found in many studies (Platt and Glimcher, 1999; Coe et al., 2002; Kobayashi et al., 2002). However, the influence of aversive outcomes has not been studied extensively.

**Figure 3:**
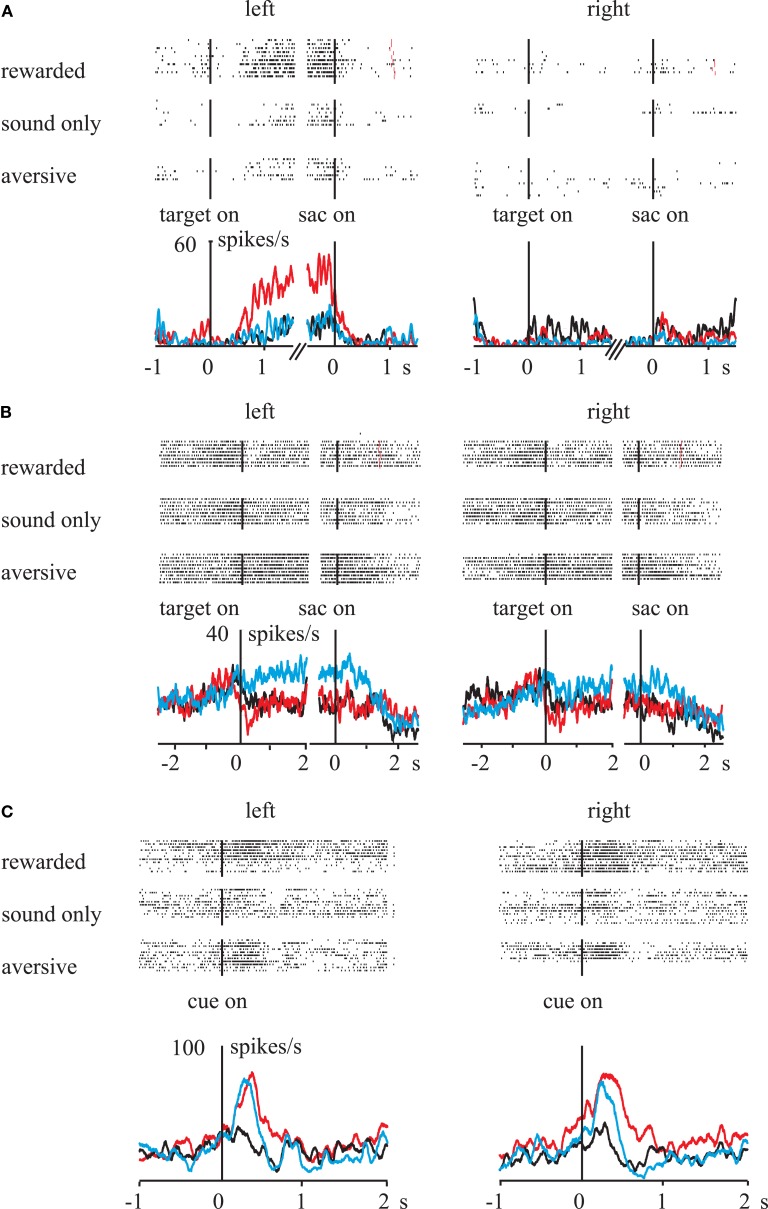
**Influences of rewarding and aversive outcomes on activity in the primate LPFC**. Raster-histograms of different types of single neurons are displayed. **(A)** The activity of this neuron increased during rewarded trials when the saccade target was presented in the left visual field. **(B)** This neuron exhibited higher delay-period activity during aversive trials when the saccade target was on the left. **(C)** The activity of this neuron increased during both rewarded and aversive trials, independent of the target cue location. Red line, rewarded trials; black line, neutral trials; blue line, aversive trials. Vertical lines indicate the onset of the events: target on, onset of the spatial cue for future saccade; sac on, saccade onset; cue on, onset of the reinforcement cue. Reprinted with permission from Neuron (Kobayashi et al., 2006).

We examined the influences of outcome value on the function of spatial working memory by recording single-unit activities in the lateral prefrontal cortex (LPFC; Kobayashi et al., 2006). Monkeys were required to remember the location of a briefly presented visual cue to perform a saccade response after a short delay. Correct responses were followed by liquid reward, air-puff avoidance, or neutral sound feedback. We found that a sizeable fraction of prefrontal neurons distinguished between rewarding and aversive outcomes. Most valence-discriminating neurons were sensitive to rewards (Figure 3A; cf. Figures 1A,B), although a small number of neurons showed activity that was preferentially modulated on aversive trials (Figure 3B; cf. Figures 1C,D). The results indicate that appetitive and aversive outcomes have independent influences on separate populations of LPFC neurons. Interestingly, a group of LPFC neurons exhibited modulation by both positive and negative reinforcers in the same direction (Figure 3C; cf. Figures 1E,F). Together, the LPFC appears to be equipped with both valence-specific and valence-non-specific reinforcement mechanisms, which would collectively contribute to outcome-based behavioral adaptation.

Another primate study examined the influence of reinforcement feedback on the LPFC and ACC (Rothe et al., 2011). LFPs were recorded while monkeys performed a problem-solving task. A correct target had to be searched by trial and error and then the correct responses could be repeated (repetition period). Error feedback caused high gamma power increases in the ACC, followed by a later increase in the LPFC during the search period. Correlations of high gamma activity were present during both the search and repetition periods, but correlations of beta power were predominant during the repetition period. Thus, feedback information appears to transfer from the ACC to the LPFC, and the functional coordination may use different LFP power bands depending on the task requirements. Evaluative signals in the ACC appear to trigger increased control by the LPFC.

There are strong and specific anatomical connections between the ACC and the LPFC, which may mediate cognitive interactions (Medalla and Barbas, 2010). The relationships between evaluative functions in the ACC and executive functions in the LPFC would account for rapid behavioral adaptation.

## Summary

Sensory processing has divergent streams for different goals: the lateral system for localizing and discriminating sensory stimuli, and the medial system for obtaining affective and motivational values. There is a wealth of evidence that the medial pain system, the core stations of which include the PAG, medial thalamus, and ACC, processes noxious inputs and generates negative affect. The medial pain system may complement the dopamine system, which processes reward value and generates prediction error signals. The PAG is thought to be involved in automatic responses such as freezing. The amygdala-OFC system plays a key role in aversive association learning. This system may enable the anticipation of harmful events based on their predictors. The amygdala-OFC system may also contribute to appetitive association learning. In addition to its role in pain perception, the ACC generates feedback signals that are triggered by behavioral errors and negative reinforcements. The feedback signals emerge as ERNs, which may reflect negative prediction errors. The ACC-LPFC connections appear to bridge the medial and lateral pathways by sending feedback signals generated in the medial pathway to control the ongoing cognitive processes in the lateral pathway.

## Perspectives

Pain and pleasure may be two sides of the same coin. How the brain treats the opposing signals is an important question that remains to be unanswered. The common currency theory provides a simplistic view that various kinds of rewards are converted into a value measure (Montague and Berns, 2002). Whether aversive learning is explained in this framework remains to be elucidated.

In addition to theoretical interest, research into the aversive system has clinical implications for pain treatment. A greater understanding of the pharmacological and physiological mechanisms underlying the aversive system is essential for the advancement of therapeutic approaches to pain (Nguyen et al., 2011).

## Conflict of Interest Statement

The author declares that the research was conducted in the absence of any commercial or financial relationships that could be construed as a potential conflict of interest.
